# Neurofibromatosis-Noonan syndrome and growth deficiency in an Iranian girl due to a pathogenic variant in *NF1* gene

**DOI:** 10.1186/s40246-023-00460-0

**Published:** 2023-02-20

**Authors:** Setila Dalili, Seyyedeh Azade Hoseini Nouri, Reza Bayat, Shahin Koohmanaee, Manijeh Tabrizi, Marjaneh Zarkesh, Alireza Tarang, Nejat Mahdieh

**Affiliations:** 1 Pediatric Diseases Research Center, Guilan University of Medical Sciences, Rasht, Iran; 2 Agriculture Biotechnology Research Institute, Agricultural Research, Education and Extension Organization (AREEO), Rasht, Iran; 3 Growth and Development Research Center, Tehran University of Medical Sciences, Tehran, Iran; 4 Cardiogenetic Research Center, Rajaie Cardiovascular Medical and Research Center, Iran University of Medical Sciences, Tehran, Iran

**Keywords:** Neurofibromatosis type 1, Neurofibromatosis-Noonan syndrome, NF1, Growth deficiency

## Abstract

**Background:**

Mutations in NF1 gene could cause allelic disorders with clinical spectrum of Neurofibromatosis type 1 to Noonan syndrome. Here, a 7-year-old Iranian girl is described with Neurofibromatosis-Noonan syndrome due to a pathogenic variant in NF1 gene.

**Methods:**

Clinical evaluations were performed along with genetic testing using whole exome sequencing (WES). The variant analysis including pathogenicity prediction was also done using bioinformatics tools.

**Results:**

The chief compliant of the patient was short stature and lack of proper weight gain. Other symptoms were developmental delay, learning disability, inadequate speech skill, broad forehead, hypertelorism, and epicanthal folds, low set ears and webbed neck. A small deletion, c.4375-4377delGAA, was found in NF1 gene using WES. This variant was classified as pathogenic according to ACMG.

**Conclusions:**

NF1 variants may show variable phenotypes among the patients; identifying such variants is helpful in therapeutic management of the disease. WES is considered as an appropriate test to diagnose Neurofibromatosis-Noonan syndrome.

## Introduction

NF1 (neurofibromatosis type 1), also known as Von Recklinghausen’s disease, is an autosomal dominant neurocutaneous syndrome with a prevalence of 1/3000 to 1/4000 births [1]. This is a multi-systemic disorder which is manifested by the involvement of central nervous system (CNS), skin, endocrine, orthopedic and gastrointestinal (GI). It is characterized by neurofibromas (simple/ plexiform), cafe-au-lait spots, axillary or inguinal freckles, Lisch nodules in the iris, optic nerve glioma, osseous lesions, and brain tumors. NF1 is diagnosed based on clinical findings with reference to “the United States National Institutes of Health (NIH)” criteria. Presence of two or more criteria out of 6 criteria of the updated version (2021) is consistent with the diagnosis of this disease and if the clinical criteria are complete, genetic analysis is not necessary.

Noonan syndrome (NS) is an autosomal dominant syndrome whose the most common presentations are short stature, facial dysmorphisms, broad forehead, triangular shape face, hypertelorism, down slanting palpebral fissure, strabismus, low set and posteriorly rotated ears, webbed neck, pectus deformity, congenital heart disease (pulmonic stenosis and hypertrophic cardiomyopathy) and cryptorchidism in male cases [2–4]. NS is caused by constitutional dysregulation in genes encoding components of the RAS/mitogen-activated protein kinase (MAPK) signaling pathway [3, 5]. Mutations in genes of Ras/MAPK signaling pathway (PTPN11, SOS1, RAF1, and KRAS) are known to cause NS phenotype [5]. The diagnosis of NS should be confirmed by finding a heterozygous pathogenic or likely pathogenic variant in one of the genes *PTPN11, SOS1, RIT1, RAF1, KRAS, SOS2, MRAS, NRAS, BRAF, RASA2, RRAS2, MAP2K1* [6–13] genes or homozygous pathogenic variants in *LZTR1* gene [13].

Different pathogenic variants of *NF1* gene may cause allelic disorders such as NF1, Watson syndrome (WS) and NFNS1 [14]. NF1 and NS are a group of clinically related disorders that happen due to mutation in RAS-MASK pathway and show phenotypical overlap [15]. In fact, NFNS1 (OMIM 601321) is a RASopathy syndrome which was first described in by Allanson et al. in 1985 [16]. They reported four unrelated patients with this syndrome. Approximately 25% of NF1 patients show also Noonan-like features. Due to similar pathophysiology, symptoms of NS may be detected in NF1 patients. The end result will be a rare condition named as NSNF1 syndrome [15, 17]. We present a 7-year-old Iranian girl clinically diagnosed with Noonan syndrome plus cafe-au-lait spots, and genetic confirmation of Noonan -Neurofibromatosis 1 syndrome (NSNF1).

## Material and methods

### Genomic analysis

This study was conducted on a seven-year-old girl referred to the outpatient endocrinology clinic at 17 Shahrivar children hospital, Rasht, Iran. A complete clinical evaluation was performed. Family history and clinical signs and symptoms were recorded. After taking informed consent, DNA was extracted from the whole blood of the patient. Whole Exom sequencing (WES) was performed; briefly, the whole coding regions were enriched by the SureSelect Human All Exon (Agilent) and sequenced using the Illumina NovaSeq 6000 Sequencing platform. The average depth was 100x. The whole exome sequencing data was analyzed in several steps: FastQC tool (version 0.11.9) was used for quality control of the reads. Trimmomatic software (version 0.36) was applied to remove the adapters. Bowtie2 (Version 2.4.0) was used to align the sequence reads to the reference genome of human (GRCh38/hg38). Realignment of insertion/deletion (indels) was done using Genome analysis toolkit (GATK). Ensembl Variant Effect Predictor tool was applied on the VCF (Variant Call Format) file for annotation of the variant.

Variants with a minor allele frequency (MAF) of more than 1% were removed according to the 1000 Genomes project (www.1000genomes.org). For more analysis, the variants were investigated and compared in the following databases: the Exome Aggregation Database (http://gnomad.broadinstitute.org), Exome Sequencing Project 6500 (http://evs.gs.washington.edu/EVS/), the Exome Aggregation Consortium database (http://exac.broadinstitute.org) and the Greater Middle East Variome Project (http://igm.ucsd.edu/gme/).

The targeted region was amplified by PCR according to a conventional protocol. Then, DNA sequencing of PCR products was performed using a BigDye termination method by sequencing analyzer of ABI3500XL model (PE Applied BioSystems, Massachusetts, USA).

### Clinical analysis

The patient was a seven-year-old girl and her chief compliant was short stature and insufficient weight gain. She has been under the supervision of a pediatrician since the age of two due to growth impairment. Extensive evaluation was done with a clinical suspicion of Noonan syndrome. She was the third live birth of the consanguineous marriage (cousin) who was born by cesarean section at 39 weeks of gestation. Birth weight, height and head circumference were 3850 g, 51 cm, and 35 cm, respectively. On the day of admission, the patient weighed 19 kg (< 5% of CDC percentile and z-score of < − 2.5). Patient’s height was 109 cm, which showed percentage of less than 5% and a z-score of − 1.3. Head circumference was equivalent to 55% of percentile. She had history of only one hospitalization due to post vaccination fever. She had global developmental delay. Speech skills were still abnormal and she had trouble saying some words. Moderate degrees of learning disability were mentioned by parents as well.

The patient did not enter school or kindergarten due to developmental delay, learning disability, and inadequate speech skill. There was no history of seizure.

**Fig. 1:**
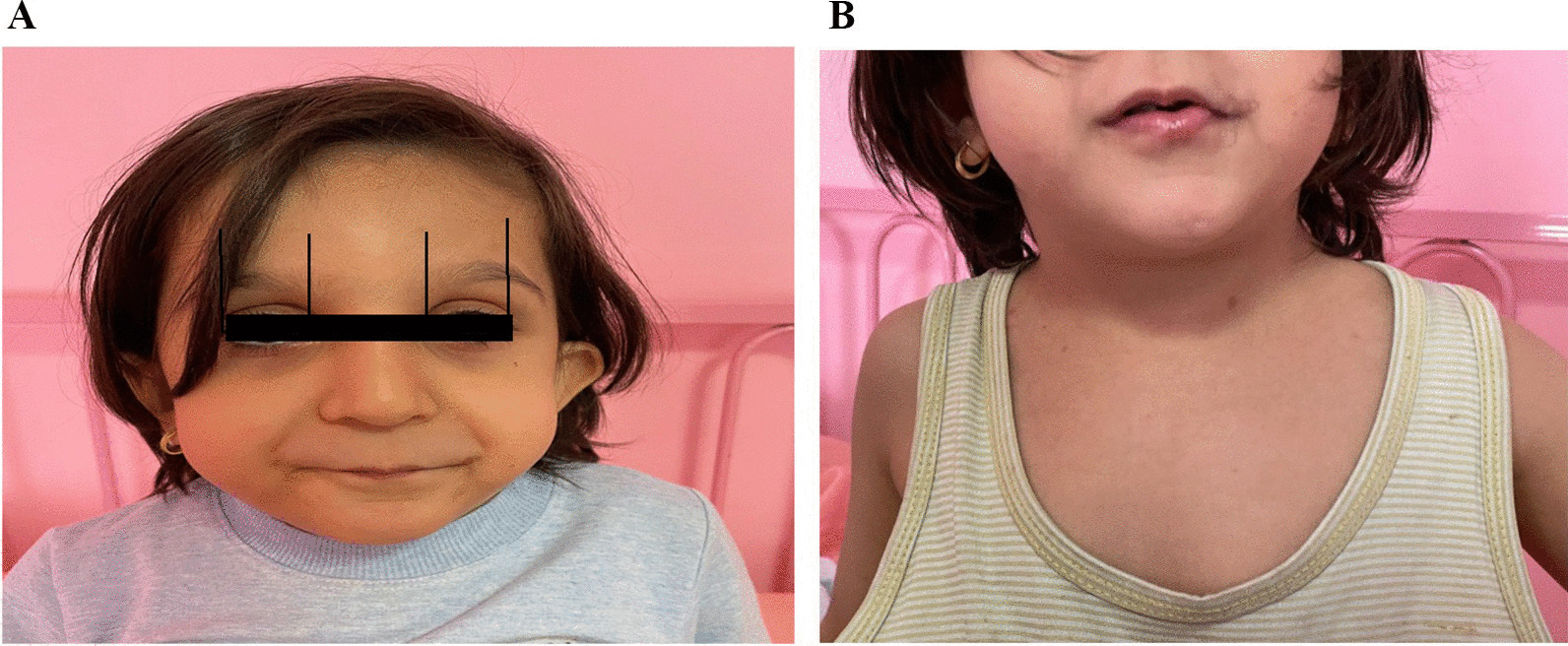
**A** Hypertelorism, low set ears, and epicanthal folds in a girl with NFNS, **B** Webbed neck

Broad forehead, hypertelorism, and epicanthal folds were evident on her face. There were bilateral low set ears (Fig. 1A) and webbed neck was observed on physical examination as shown in Fig. 1B.

**Fig. 2:**
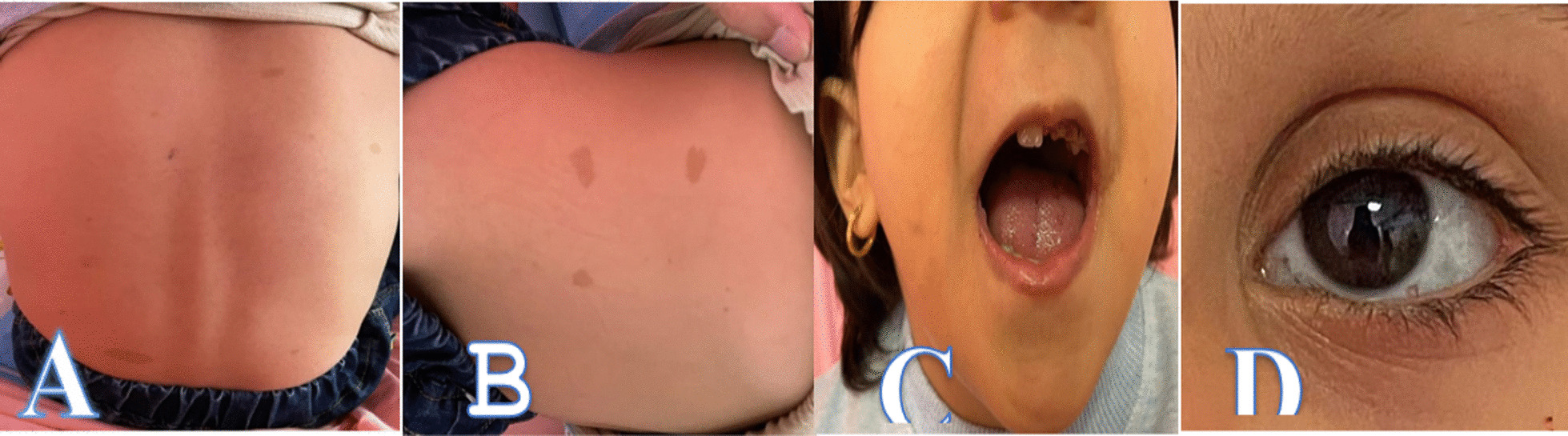
**A, B** Multiple cafe-au-lait spots with various sizes on the trunk. Dark brown hyperpigmented macules on the dorsal surface of tongue (**C**) and sclera (**D**)

Multiple cafe-au-lait spots in different sizes were seen on the trunk, back, and proximal limbs. Some of them were up to 4 cm (Fig. 2A, B).

She also had multiple dark brown hyperpigmented macules on the dorsal surface of the tongue, the buccal surface, and the sclera (Fig. 2C, D). Her parents mentioned that cafe-au-lait spots appeared in the first months after birth and are gradually increased in number and size. She had no axillary or inguinal freckles. A thorough examination of the patient revealed no neurofibroma. No chest deformity, scoliosis or other skeletal problems were evident in examination. The distance between the two nipples was normal. Echocardiography that was performed in neonatal period because of a cardiac murmur was consistent with vulvar and supra vulvar pulmonary stenosis, and mild tricuspid regurgitation. These findings were still present in serial and recent echocardiography. Patient had grade 2–3 systolic ejection murmurs on the left second intercostal, below the margin of sternum. Abdominal exam, blood pressure and, other vital signs were normal.

Routine laboratory tests (Complete blood count, routine biochemistry, and urine analysis) were within the normal limits. The celiac screening antibodies were negative and thyroid function tests were normal. Abdomino-pelvic sonography revealed no abnormality. Chest radiography was normal as well. On the left wrist radiography, the bone age was equivalent to the chronological age (7 years old).

Brain MRI revealed no pathological finding. The visual acuity was normal. Ophthalmoscopy and slit lamp examination revealed no Lisch nodule, optic disc atrophy, or any choroid abnormality. Audiometry performed and did not show any degrees of hearing loss. Her parents and siblings were healthy and without any freckle or cafe-au-lait spots.

### Genetic results and genotype–phenotype architecture

An accurate diagnosis of a seven-year-old girl was established using WES. The results showed a small deletion variant in *NF1* gene, c.4375-4377delGAA (p.Glu1459del). In silico analysis showed that this variant has deleterious effects. ACMG guideline was applied and the variant was classified as a pathogenic variant.

A literature search showed that 3890 variants have been reported in *NF1* gene (https://www.hgmd.cf.ac.uk). HGMD data shows that missense/nonsense, small indels and splicing variants were more frequent than other types of variant. More than 97% of variants have been reported to cause NF1 phenotypes. Less than 3% of *NF1* variants have been described with other phenotypes such as Neurofibromatosis-Noonan syndrome, phaeochromocytoma, multiple congenital anomalies, autism spectrum disorder, epilepsy, intellectual disabilities and cardiovascular malformations. Of 26 variants found in *NF1* associated with NFNS1, 13 were pathogenic variants (10 missenses and 3 nonsenses), 2 were splice variants, 9 were small deletions and 2 were large deletions (data not shown). p.Leu1196Phe has been reported in patients with Noonan and NFNS1 syndrome.

## Discussion

NSNF1 is a rare autosomal dominant hereditary disease with manifestations of both NF1 and NS. NFNS1 is estimated to have a higher frequency than 25% of neurofibromatosis 1 patients [18] because of being misdiagnosed as classic NF1 or NS. Patients with NF1 may have a Noonan like facial features. Noonan syndrome may present with café-eu-lait macules, and if this finding, especially a large number (> 6) or size (> 5 mm) of them, which fulfills a diagnostic criteria for NF1, therefore it is better to undergo a genetic evaluation in terms of accompaniment with NF1 [5]. Genetic testing using next generation sequencing could provide an accurate diagnosis. In a study, the re-examination of 94 NF1 patients showed that 13 of them also had NS criteria [19]. We describe a case of NFNS1 with short stature due to a small deletion variant in NF1 gene, c.4375-4377delGAA. A 7-year-old Iranian girl was admitted for short stature, numerous café-au-lait spots, webbed neck, low-set ears and broad forehead.

Of more than 3 thousands reported *NF1* variants, only 26 ones have been reported to cause NSNF1; the majority of these variants are missense followed by small indels. Type of variant may have an effect on phenotype as we have reported such a phenomenon about *MYO15A* gene and *MYBPC3* gene [20, 21].

A case of Noonan-Neurofibromatosis 1 has been reported in Iran so far by Yazdizadeh et al., that was accompanied by giant cell Granuloma [22]. Isik et al. presented a seven-year-old girl with typical clinical features of NF1 and short stature and abnormal genital appearance (Plexiform neurofibromas) and, after a molecular analysis, a novel heterozygous c.3052_3056delTTAGT was found [23].

The main causes of growth problems in NF-NS are skeletal deformities and nutritional deficiencies [19]. The mechanism of GH deficiency in *NF1* patients is still unknown, although it is known that GH treatment increases the final height in NS cases. However, no consensus has been reported regarding administration of growth hormone for NFNS patients. Since the administration of growth hormone (GH) can increase the size of nodules in NF1 patients [19, 24], GH was not administered to our patient. So far, one case of growth hormone administration in NFNS patients has been reported [24]. Pulmonary artery stenosis is a common cardiac defect in NSNF1 [25] which was also detected in our case.

Unlike adult patients, the NIH criteria in children are not so specific and sensitive [1]; because a variety of clinical signs of NF1 syndrome may occur over time. By the age of 20, the clinical manifestations and clinical criteria of NF1 will usually be completed. Because of delayed onset of symptoms and completion of criteria, clinical diagnosis is difficult in childhood and commonly should be confirmed with genetic testing [1]. Genetic testing is strongly recommended in a child presenting Noonan symptoms in companion with even an incomplete NF1 criteria [1]. The present case had typical manifestations of NS. Moreover, there were obvious cafe-au-lait spots in her skin, tongue and sclera, which raised clinical suspicion of Rasopathy or NSNF1 syndrome. Cafe-au-lait spot or macule (CALM) as the first manifestation of NF1 is well-defined; 0.2–30 cm in diameter, light brown-medium-dark brown lesions could be observed on all over the body surface except for the scalp, palms, and soles. The borders may be smooth or irregular. Such lesions may appear in the first years of life or even at birth [26].

Neurofibromas are benign nerve sheet tumors developed in the skin and subcutaneous tissue [1]; although this patient had no evidence of neurofibroma yet, it is possible that they occur over time. Stenenson et al. reported involvement of a mother and 2 offspring by NFNS who had multiple cafe-au-lait spots and relative macrocephaly without any neurofibromas [27]. Brain MRI of NSNF1 cases could display focal areas of signal intensity in deep white matter and basal ganglia or corpus callosum, areas of hyper intensity with no contrast enhancement, optic nerve or optic pathway glioma, progressive sphenoid wing dysplasia, lambdoid suture defects, dural calcification at the vertex, and rarely moya-moya phenomenon [17, 28–30]. However, the brain MRI of the present case was reportedly normal. NF1 patients show an increased prevalence of benign tumors and some certain malignancies [31]. To prevent malignancy, the changing in the size of lesions or the start of pain in a neurofibroma or the infiltration of the adjacent structures should be checked [32]. In these cases, fludeoxyglucose (FDG) positron emission computerized tomography is recommended [31]. Some malignancies have also been reported in NF1 patients including gastrointestinal stromal tumor, osteosarcoma, rhabdomyosarcoma [33], thyroid, bone, ovary and breast cancer, renal angiomyolipoma, juvenile pilocytic astrocytoma, myeloproliferative disease, acute lymphoblastic leukemia and melanoma [34]. However, the incidence of malignancies is unclear in Noonan spectrum [33, 35]. Annual visual examination, ophtalmoscpy and audiometry are recommended. Evaluations for signs of precocious puberty and malignancy are also mandatory [31–33]. The first degree relatives of the patients should undergo cascade screening by genetic testing and ophthalmoscopic examinations.

## Conclusion

NFNS1 may have various symptoms and signs in different patients; multiple cutaneous lesions (> 6) associated with NS syndrome suggest the clinician to check for an associated genetic disorder. The presentation of our case reinforces the importance of careful examination for the clinical features of other RASopathies in patients with Noonan syndrome. Finally, genetic testing is strongly recommended in any case with Noonan syndrome whose clinical findings are consistent with rasopathies (even if not fulfilling the complete diagnostic criteria). This is of great help in timely diagnosis and appropriate later follow-up.

## Abbreviations

WES: Whole exome sequencing
CNS: Central nervous system
GI: Gastrointestinal
NS: Noonan syndrome
MAPK: RAS/mitogen-activated protein kinase
WS: Watson syndrome
NSNF1: Noonan-Neurofibromatosis 1 syndrome
GH: Growth hormone
CALM: Cafe-au-lait spot or macule
FDG: Fludeoxyglucose
